# Antimicrobial Resistance: The Major Contribution of Poor Governance and Corruption to This Growing Problem

**DOI:** 10.1371/journal.pone.0116746

**Published:** 2015-03-18

**Authors:** Peter Collignon, Prema-chandra Athukorala, Sanjaya Senanayake, Fahad Khan

**Affiliations:** 1 ACT Pathology, Canberra Hospital, Australian National University, Garran, Australia; 2 Canberra Clinical School, Australian National University, Garran, Australia; 3 Arndt-Corden Department of Economics, Australian National University, Acton, Australia; 4 School of Environment and Development, University of Manchester, Manchester, England; 5 Australian National University, Garran, Australia; 6 Canberra Hospital, Garran, Australia; University of Calgary, CANADA

## Abstract

**Objectives:**

To determine how important governmental, social, and economic factors are in driving antibiotic resistance compared to the factors usually considered the main driving factors—antibiotic usage and levels of economic development.

**Design:**

A retrospective multivariate analysis of the variation of antibiotic resistance in Europe in terms of human antibiotic usage, private health care expenditure, tertiary education, the level of economic advancement (per capita GDP), and quality of governance (corruption). The model was estimated using a panel data set involving 7 common human bloodstream isolates and covering 28 European countries for the period 1998–2010.

**Results:**

Only 28% of the total variation in antibiotic resistance among countries is attributable to variation in antibiotic usage. If time effects are included the explanatory power increases to 33%. However when the control of corruption indicator is included as an additional variable, 63% of the total variation in antibiotic resistance is now explained by the regression. The complete multivariate regression only accomplishes an additional 7% in terms of goodness of fit, indicating that corruption is the main socioeconomic factor that explains antibiotic resistance. The income level of a country appeared to have no effect on resistance rates in the multivariate analysis. The estimated impact of corruption was statistically significant (p< 0.01). The coefficient indicates that an improvement of one unit in the corruption indicator is associated with a reduction in antibiotic resistance by approximately 0.7 units. The estimated coefficient of private health expenditure showed that one unit reduction is associated with a 0.2 unit decrease in antibiotic resistance.

**Conclusions:**

These findings support the hypothesis that poor governance and corruption contributes to levels of antibiotic resistance and correlate better than antibiotic usage volumes with resistance rates. We conclude that addressing corruption and improving governance will lead to a reduction in antibiotic resistance.

## Introduction

Antibiotic resistance is a growing international problem. This has led to increasing numbers of serious infections that are very difficult, or sometimes impossible to treat. Increasing resistance involves nearly all bacteria that infect people, including very common ones such as *Escherichia coli* and *Staphylococcus aureus*. People with infections caused by resistant isolates have much higher death rates as well as increased complications and suffering. Antimicrobial resistance also has important economic costs such as the use of more expensive drugs, waste and increasing the length of hospital stays [1–6].

Antibiotic resistance develops and spreads wherever antibiotics are used, not only in medical facilities but also in the community. Poor infection control, poor water sanitation and poor hygiene all facilitate the spread of resistant bacteria from person to person. The majority of antibiotic usage worldwide is in food animals [7–9]. This usage leads to the development of resistant bacteria, which spread to people via the food chain and/or water [9–13].

The general perception of antibiotic resistance is that it is almost entirely related to the amounts of antibiotics used, not only in the broad sense of comparative usage by different countries but also in individuals [14–17]. However, the available empirical evidence suggests that these two variables are not perfectly correlated at national levels and across countries [18–20]. We believe that other factors are as important, or even more important, to account for the variations in resistance observed between regions and countries. In particular, we wished to look at the contribution of corruption.

We postulate that a fuller understanding of antibiotic resistance requires a unified approach which combines not only the amount of antibiotic usage but also other factors that may impact on the quality of antibiotic usage, control and the spread of resistant bacteria at national levels. We therefore undertook a multivariate analysis of determinants of antibiotic resistance in bacteria causing bloodstream infections throughout Europe.

## Model and Data

For the empirical analysis, we used a newly-constructed panel data set covering 28 countries in Europe over the period 1998–2010 (full supporting information are available on the web; S1 Table). This dataset enabled us to examine both intra-country and inter-country variation in antibiotic resistance in terms of antibiotic usage and four other national-level variables which are likely to impact on the quality of antibiotic usage and the spread of resistant bacteria. The latter variables are: private health care expenditure, tertiary education, the level of economic advancement and the quality of governance (institutional quality, in particular, corruption).

The multivariate regression model used for explaining antibiotic resistance is,

$$ABR_{i}{,}_{t}=\beta_{0}+\;\beta_{1}ABU_{i}{,}_{t}\;+\beta_{2}GOV_{i}{,}_{t}\;+\;\beta_{3}PHE_{i}{,}_{t}+\;\beta_{4}PGDP_{i}{,}_{t}+\;\beta_{5}TED_{i}{,}_{t}+\;\beta_{6}AGR_{i}{,}_{t}+\;\mu_{i}+\;\gamma_{t}+\;\varepsilon_{i}{,}_{t}$$

where, the dependent variable (ABR—Antibiotic Resistance Rate) is the rate of resistance to antibiotics measured in bacteria causing bloodstream infections, i = 1, 2, …, N is the subscript representing country, and t = 1, 2, …, T represents the time unit in years.

The explanatory variables, with the expected sign of the regression coefficient in brackets, are

*ABU* quantity of antibiotic usage in people expressed as Defined Daily Doses (DDD) (+),

*GOV* the quality of governance, defined as the control of corruption in society measured on a discrete scale from zero to six (-),

*PHE* private health expenditure as a percentage of national income (+),

*PGDP* per capita gross domestic product in constant (2000) price (in natural logarithms) (-),

*TED* percentage of tertiary educated people in total population (-),

*AGR* percentage of people employed in the agriculture sector out of total employment (+)

μ_i_ Country-specific and time-invariant fixed factors.

γ_t_ Time Effects (i.e. binary variables for each year except for the first year) to capture time varying common shocks or global trends, and

ε_i,t_ is the idiosyncratic error term that captures all other determinants of ABR

*ABU* and *GOV* are the main explanatory variables that we are interested in comparing as determinants of *ABR*. *ABU* is the variable commonly associated with antibiotic resistance and which is assumed to have a positive impact on antibiotic resistance. *GOV* captures the degree to with the rule of law and social norms are honored in a given country. This will not only affect the operations of government, infrastructure and social services but also the private sectors and the quality of medical practices. The other variables are the control variables which are simultaneously correlated with the main explanatory variables of interest as well as the dependent variables. It is important to include these variables in the model to mitigate omitted variable bias. *PHE* may have a positive impact on antibiotic resistance if greater access to private medical sector leads to increased antibiotic usage may have a negative impact on antibiotic resistance, if economic prosperity is positively related with greater public understanding of health risk, better housing and on the availability of better public infrastructure. *TED* would have a negative impact on antibiotic resistance if higher education levels result in greater public understanding of antimicrobial-resistance health risks.

Europe was chosen for estimating the model because it is the only region where good data for all of our parameters are available across multiple countries. Our panel data set covered 28 countries in Europe over the period 1998–2010. The countries covered are Austria, Belgium, Bulgaria, Cyprus, Czech Republic, Denmark, Estonia, Finland, France, Germany, Greece, Hungary, Iceland, Ireland, Italy, Latvia, Lithuania, Luxembourg, Malta, Netherlands, Norway, Poland, Portugal, Slovakia, Slovenia, Spain, Sweden, and United Kingdom. The country- and time-coverage of the analysis are dictated by the nature of availability of data on the dependent variables (ABR). These data on antimicrobial resistance were compiled from EARS-Net Database of the *European Centre for Disease Prevention and Control* [18]. This database contains resistance data for bacteria that are the most important and frequently encountered causes of bloodstream infections. In December 2008 it had data on more than 700,000 invasive isolates.

In this study, we looked at 25 pathogen/antibiotic combinations which are grouped under seven pathogen classes. The pathogen classes and the relevant antibiotics are listed as follows:

*Streptococcus pneumoniae* resistance to penicillins (PNSP) and macrolides (MNSP)
*S. aureus* resistance to methicillin (MRSA) and rifampicin
*Pseudomonas aeruginosa* resistance to amikacin, aminoglycosides, carbapenems, ceftazidime, fluoroquinolones and piperacillin/tazobactam
*Klebsiella pneumoniae* resistance to 3^rd^ gen cephalosporins, aminoglycosides, carbapenems and fluoroquinolones.
*E. coli* resistance to 3^rd^ gen cephalosporins, aminoglycosides, aminopenicillins, carbapenems and fluoroquinolones.
*Enterococcus faecium* resistance to aminopenicillins, high-level gentamicin and vancomycin
*Enterococcus faecalis* resistance to aminopenicillins, high-level gentamicin and vancomycin

Data for ‘S. *pneumoniae*’ group of resistances are not available for Greece, while data for ‘*P. aeruginosa*’ *and* ‘*K. pneumoniae*’ are not available for Slovakia. After allowing for these data gaps, the regression estimates are based on 161 to 298 observations.

Data on antibiotic usage are from *European Surveillance of Antimicrobial Consumption* (ESAC) Yearbook 2009 [19]. In this source, antibiotic usage is measured as total outpatient antibiotic use expressed in Defined Daily Doses (DDD) per 1000 inhabitants per day. We extrapolated the data to 2010 using linear extrapolation. The data on the other explanatory variables are extracted from the *World Development Indicators* database of the World Bank [21].

*PGDP* is per capita gross domestic product (*GDP*) converted to constant year 2000 international dollars using purchasing power parity rates. Tertiary school enrolment is measured in terms of the ratio of total enrolment in tertiary educational institutions, regardless of age, to the population of the age group that officially corresponds to the level of education shown. Private health care expenditure (PHE) includes direct household (out-of-pocket) spending, private insurance, charitable donations, and direct service payments by private corporations. We use this as a percentage of total Gross Domestic Product in order to normalise for the size of the economy. Agriculture share of employment (AGR) is based on both public and private employment in agriculture (which corresponds to ISIC revision 2), and receive any kind of a remuneration. These variable definitions are from World Development Indicators [21].

In this paper, we specifically focus on the quality of governance in comparison to antibiotic usage as determinants of antibiotic resistance, while controlling for the other possible determinants. We measure the quality of governance in terms of the control of corruption, which is the most ubiquitous manifestation of governance quality in policy debates [23–24]. Henceforth, we use the terms “quality of governance” and “control of corruption” synonymously. The empirical analysis is based on a panel data set compiled by combining data on antibiotic usage and resistance in people in Europe with data on other variables extracted from other relevant sources that are widely employed in empirical economic analysis.

The indicator governance (*GOV*) comes from the International Country Risk Guide developed by the Political Risk Services Group [26]. This indicator measures the quality of governance in terms of control of corruption based on subjective assessment by experts. The indicator runs on a seven point scale from 0–6 with higher values representing less corruption (or a higher control over corruption). The major advantage of this indicator compared to the other alternative measures of the quality of governance is that data are available for all years of the time period covered in our analysis. In experimental runs we used three other alternative indicators of governance (rule of law, government effectiveness, and control of corruption) from the World Bank’s World Governance Indicators database [22] The results were comparable in the Pooled Ordinary Least Squares (POLS) estimation. However, we were not able to use these indicators in fixed effect (FE) and system Generalized Method of Moments (system GMM) estimations because of missing data for many years.

Table 1 reports the means, standard deviations, minimums, maximums, the number of countries as well as observations for all the variables in our estimation sample. The descriptive statistics reveal a substantial variation in all the variables, making it suitable for a cross-national investigation. For instance, antibiotic resistance varies from a minimum of close to 0 (for Sweden in 1998) to a maximum of 39 percent (for Greece in 2007). Similarly antibiotic usage also varied significantly from a minimum of 10 *DDD* (for Netherland in 2004) to a maximum of 45 *DDD* per day per 1000 inhabitants (for Greece in 2007). According to the country-level data (not reported here for brevity but available in S1 Table), Greece records the highest private health expenditure as a proportion of GDP in our group of countries, while Luxembourg has the lowest. GOV indicates that the country with the best governance is Finland; on the other hand, Latvia had the lowest score throughout a substantial part of our time coverage. The most prosperous country in our group in terms of GDP per capita is Luxembourg while the poorest one is Bulgaria. Bulgaria also recorded the highest employment share of agriculture, with the UK at the other end of the spectrum. Finally Finland has the highest gross tertiary enrollment, while Luxembourg has the lowest.

**Table 1:** Descriptive Statistics.

Variables	Mean	Minimum	Maximum	Countries	Observations
ABR	17.03 (7.87)	0	38.96	28	247
ABU	19.61 (6.36)	9.7	45.2	28	266
GOV	3.94 (1.20)	2	6	28	304
PHE	2.03 (0.68)	0.43	4.01	28	308
PGDP	26405(11739)	6533	74114	28	308
TED	54.86 (17.76)	9.80	95.07	28	307
AGR	6.52 (4.87)	1.1	26.2	28	308

Note: Standard Deviations are reported in parentheses.

## Estimation Methodology

The model is initially estimated using the Pooled Ordinary Least Squares (POLS) method, which estimates the equation excluding the country—specific fixed factors (μ_i_). However, ignoring country-specific fixed effects could cause omitted variable bias, because country-specific and time-invariant factors might be correlated with both ABR and the explanatory set. These factors include geography, historical experience, legal origin, ethno linguistic fragmentation and culture. We therefore re-estimated the model using the fixed effects (FE) technique. This technique involves estimating the equation after demeaning the variables to purge possible country-specific fixed effects in order to isolate the ‘within country’ relationship.

While the FE estimation technique addresses possible endogeneity arising from ignoring country specific fixed effects, it does not address two other sources of endogeneity—potential reverse causality from the dependent variable to the explanatory variables, and possible error in the measurement of the variables. For instance it is plausible that higher ABR can cause overuse of antibiotics rather than causality running in the direction we have assumed. In addition it is obvious that the construction of our variables (especially ABR, ABU and GOV which are the main variables of interest) may suffer from a measurement error. Both types of endogeneity lead to biased and inconsistent estimates for the coefficients. When the explanatory variables are potentially endogenous, we need to be cautious in inferring causality from the estimated coefficients.

We therefore use the system Generalized Method of Moments (system GMM) technique as a robustness check. This technique consistently estimates the equation even in the presence of endogenous explanatory variables and measurement error, and thus allows us to be more confident in inferring causality [30–31]. The system GMM technique is typically used in the empirical literature to consistently estimate an equation that contains the lagged dependent variable in the explanatory set. Therefore we augment the equation by including lagged ABR on the right hand side in order to estimate it through system GMM. This has the added advantage of taking into account potential persistence in ABR—it is quite conceivable that past antibiotic resistance is an important determinant of present antibiotic resistance.

System GMM generates a set of internal instruments for the right hand side variables which are uncorrelated with the error term. This set consists of the appropriate number of lags of the right hand side variables as instruments in the differenced version of the original equation and the first differences (and the appropriate number of lagged first differences) as instruments in the original level equation. The validity of the instrument set can be tested through a Hansen test of over-identifying restrictions. However, this test is weakened if the number of instruments is large relative to the number of observations, and works best if the number of time periods in the panel is small [32]. Therefore we use observations occurring every second year rather than annually when estimating through system GMM.

Note that the unit of measurement used varies among the variables employed in our analysis: *ABU* is measured in *DDDs*; *ABR*, *PHE* and *TED* are percentages; and *GOV* is an index (0 to 6) and PGDP is in thousand dollars (expressed in logs). Therefore, the regression coefficients are not directly comparable. We therefore report results using the beta coefficients to facilitate comparing the relative impact of each explanatory variable on the dependent variable. The beta coefficient places all regression coefficients on a comparable basis by measuring the impact of change in a given explanatory variable on the dependent variable, using the standard deviation as the common denominator.

## Results

Our results support the crucial role of governance in determining antibiotic resistance across these countries. When we use the simplest of our estimations, only 28 percent of the total variation in antibiotic resistance (adjusted R^2^ = 0.28) is attributable to variation in antibiotic usage in people. When the time effects are also included to take into account possible shocks or global trends (not captured by the other explanatory variables) impacting antibiotic resistance uniformly across all countries, the regression explains 33 percent of the total variation in antibiotic resistance. Once the control of corruption indicator is included as an additional explanatory variable, 63 percent of the total variation in antibiotic usage is explained by the regression. The complete multivariate regression only accomplishes an additional 7 percentage point in terms of goodness of fit, indicating that corruption is the main socioeconomic factor that explains antibiotic resistance. The income level of a country appeared to have no effect on resistance rates when we also controlled for the quality of governance in the multivariate analysis.

**Fig 1:**
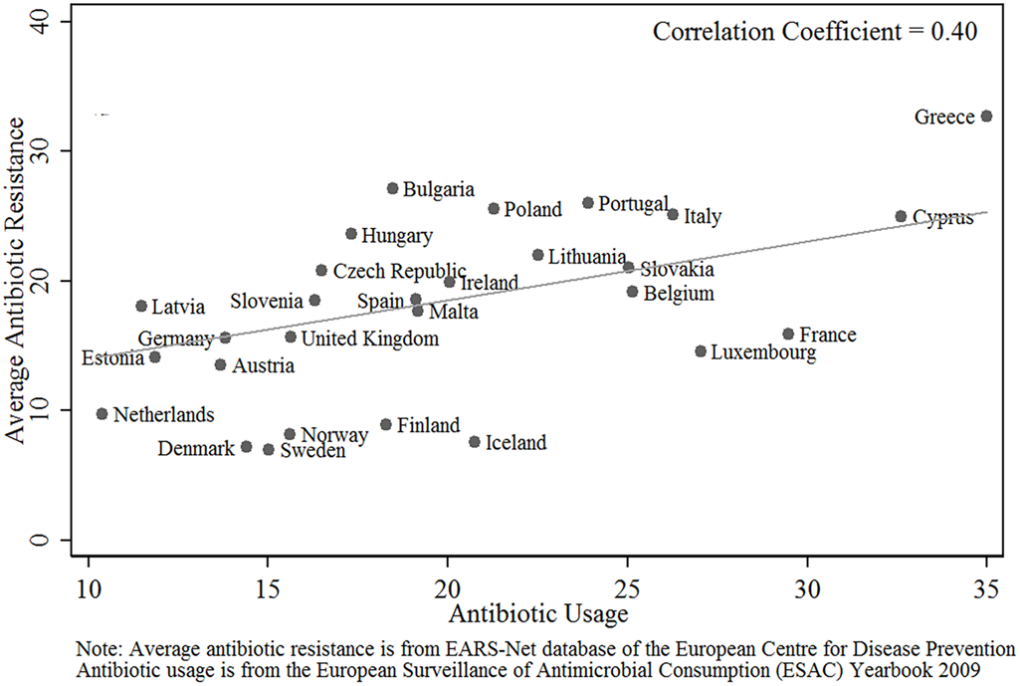
‘Average Microbial Resistance’ against ‘Antibiotic Use.’

**Fig 2:**
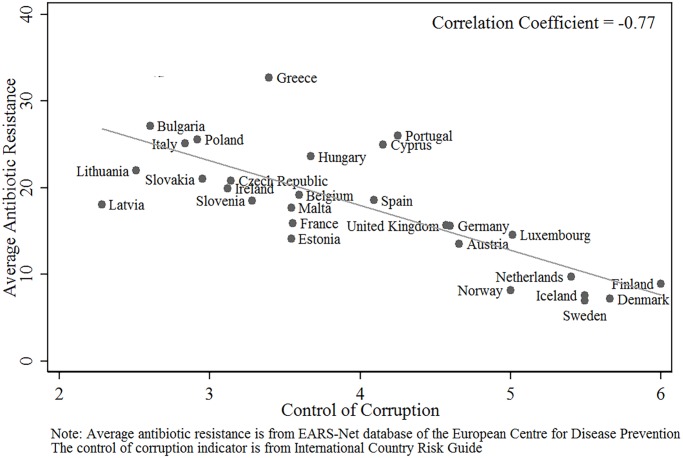
‘Average Microbial Resistance’ against ‘Control of Corruption.’

To aid interpretation of econometric estimates, the pair-wise correlation matrix for the variables is reported in Table 2. The bivariate relationship between antibiotic resistance and the two key variables of interest (antibiotic usage and governance) are plotted in Figs. 1 and 2.

**Table 2:** Pair-wise Correlation Coefficients.

	ABR	ABU	GOV	PHE	PGNI	TED	AGR
ABR	1.00						
ABU	0.53	1.00					
GOV	-0.71	-0.28	1.00				
PHE	0.47	0.35	-0.18	1.00			
PGDP	-0.39	0.05	0.52	-0.23	1.00		
TED	-0.11	-0.17	0.02	0.15	-0.07	1.00	
AGR	0.47	0.23	-0.38	0.28	-0.59	0.11	1.00

The simple correlation of each explanatory variable with antibiotic resistance is consistent with our expectations for the expected signs of the estimated coefficients. Governance is negatively correlated with *ABR*; in fact, the magnitude of this correlation is the largest compared to all other variables. Gross tertiary enrollment has the smallest pair-wise correlation with *ABR*.

Figs. 1 and 2 are a graphical illustration of the simple bivariate relationship of ABR with antibiotic usage and governance, respectively. The figures, together with Table 2, indicate that governance is potentially as important a determinant of antibiotic resistance as is antibiotic usage in people.

The estimation results are reported in Table 3. The first column corresponding to each estimation technique is the point of departure of our analysis from the conventional thinking that attributes increasing antibiotic resistance solely to antibiotic usage; this column contains the regression results from a standard bivariate regression of antibiotic resistance to antibiotic usage (also controlling for time effects). The next column for each technique then included control of corruption as an additional variable to the specification, which allows testing for our proposition that governance is just as important a determinant, if not more, of antibiotic resistance compared to antibiotic usage. Finally the third column for each technique contains the regression results for the complete multivariate model that we have postulated.

**Table 3:** Regression Results for Average Antibiotic Resistance.

	(1)	(2)	(3)	(4)	(5)	(6)	(7)	(8)	(9)
Independent Variables	OLS	OLS	OLS	OLS	FE	FE	SGMM	SGMM	SGMM
ABU	0.51(0.12) ***	0.36(0.09) ***	0.29(0.09) ***	0.25(0.14)*	0.16(0.11)	0.12(0.11)	0.08(0.32)	0.32(0.07) ***	0.07(0.11)
GOV		-0.56(0.07) ***	-0.37(0.10) ***		-0.25(0.12)**	-0.22(0.12) *		-0.55(0.11) ***	-0.65(0.24) ***
PHE			0.20 (0.06) ***			-0.08(0.16)			0.22(0.07) ***
PGDP			-0.17(0.08)**			-0.02(0.45)			0.06(0.16)
TED			-0.11(0.04) ***			-0.01(0.18)			-0.07(0.07)
AGR			0.13(0.17)			-0.54(0.54)			-0.02(0.15)
ABR_t-2_							0.31(0.10) ***	0.18(0.14)	0.15(0.16)
Countries	28	28	28	28	28	28	28	28	28
Observations	242	242	242	242	242	242	99	99	99
T.E included	Yes	Yes	Yes	Yes	Yes	Yes	Yes	Yes	Yes
p-value of ‘ABU = -Cor’		0.15	0.61		0.65	0.61		0.08	<0.01
Adjusted R^2^	0.33	0.63	0.70	0.80	0.81	0.81			
R^2^(within)				0.23	0.27	0.28			
Instruments							28	31	35
Sargan test p-value							0.21	0.58	0.71
Hansen J test p-value							0.47	0.60	0.79
AR(2) test p-value							0.01	0.50	0.56
Wald chi-sq statistic							74.36	227.99	845.02
Wald chi-sq p-value							<0.01	<0.01	<0.01

Notes:

The standardized (beta) regression coefficients are reported in the table. TE refers to the set of time dummy variables i.e. the time effects. The estimated coefficient for the constant term and TE are not reported. ‘ABU = -Cor’ refers to the F test for equality of the magnitude for the coefficient of *Antibiotic Usage* and *Control of Corruption*. *P* value is the probability of obtaining the observed test statistic. We reject the null hypothesis if the p-value is less than the level of statistical significance at which the test is conducted. For Pooled OLS and Fixed Effects regressions, standard errors clustered by countries are reported in parenthesis. Annual observations are used from 1998–2010. The Adjusted R^2^ value of the regression in Column (1) without including the time effects is 0.28. The R^2^ in Columns (4), (5) and (6) refers to the coefficient of determination from estimation of the equivalent Least Squares Dummy Variable Model (LSDV).

For System GMM, Windmeijer-Corrected Robust standard errors from the two-step GMM estimation are reported in parenthesis. Observations at 2 year intervals used from 1998–2010. Orthogonal forward deviations are used to purge fixed effects. The main explanatory variables (*Antibiotic Usage* and *Control of Corruption*) are treated as endogenous and instrumented by the collapsed matrix of all available lags.

*Significant at the 10% level

**Significant at the 5% level

***Significant at the 1% level

Focusing on the complete multivariate regression (Column 3), the coefficients of all variables have the expected sign and, except that of the employment share of agriculture, are statistically significant at the one percent or five percent level. In terms of magnitudes of the coefficients, the governance variable has the greatest association with antibiotic resistance; a one unit improvement in the indicator is estimated to lower antibiotic resistance by 0.4 units. A similar reduction in antibiotic usage only reduces resistance by 0.3 units. As for the other variables which are statistically significant, a one unit increase in growth in tertiary enrollment brings about a reduction of 0.1 units in *ABR*, while a one unit increase in *GDP* per capita and decrease in private health expenditure (as a proportion of GDP) are each associated with a reduction of 0.2 units in *ABR*.

The FE results reported in Columns [3–6], however, present a different picture and suggest that POLS estimates must be treated with caution. Once country specific fixed factors are taken into account, except for governance (our key variable of interest), all other variables lose their explanatory power in determining antibiotic resistance. Each FE regression explains more than 80 percent of the total variation in antibiotic resistance in terms of the Adjusted R^2^: adding additional variables into the specification practically makes no difference, indicating the central role of fixed country characteristics such as culture and geography. Interestingly, when fixed country characteristics are controlled for, governance is still a statistically significant determinant of antibiotic resistance. The estimated coefficients suggest that, in the within country context, a one unit improvement in the corruption score is associated with a 0.2 unit reduction in ABR.

The system GMM estimates are given in Columns 7–9. The results suggest a causal relationship between our variables of interest after addressing all possible sources of endogeneity. The diagnostics indicate that the model has been adequately estimated. The p-value of the Hansen test of over-identifying restrictions indicates that the instrument set, which identifies the exogenous variation in the explanatory variables, is valid. Moreover, the p-value is not unrealistically high, which would have suggested an over-fitting bias. The p-value of the AR (2) test also indicates that there is no second order serial correlation in the residuals, a necessary condition for consistent estimation. An unrealistically high p-value is a symptom of instrument proliferation in which case the Hansen test is weakened [32].

Control of corruption remains the most important determinant of antibiotic resistance according to the system GMM results. The coefficient of *ABU* is estimated with precision only in the second regression, and even then it is significantly smaller in magnitude compared to the coefficient of *GOV* (as indicated by the p-value of the F-Test for equality of the coefficients). In the complete multivariate model (Column 9), the coefficient of *ABU* is not statistically significant even at the 10% level. In contrast, the estimated impact of *GOV* is statistically significant at the one percent level, and the coefficient indicates that an improvement in the “control of corruption” indicator by one unit reduces antibiotic resistance by approximately 0.7 units. Interestingly the causal impact of private health expenditure is also estimated with precision: a one unit reduction reduces ABR by 0.2 units. The system GMM results also suggest that antibiotic resistance in the previous period does not affect present resistance, and thus ABR is not a persistent variable.

## Discussion

The results of our empirical analysis show that factors other than antibiotic usage are potentially very important in explaining the different levels of resistant bacteria seen in different countries (Figs. 1 and 2). The regression with antibiotic usage in people as the only explanatory variable appears to explain only 33 percent of the total variation in antibiotic resistance seen across countries in Europe (including a 5% contribution of time effects). Once the “control of corruption” indicator is added, 63 percent of the total variation in antibiotic usage is now explained by the regression. It is of note that the GDP per capita was poorly correlated to the amount of resistance seen. These findings challenge the general perception that antimicrobial resistance is predominantly a reflection of just poverty and antimicrobial usage in people.

Private health expenditure was also an important factor. The higher the percentage of private health expenditure in a country, the larger was the degree of antibiotic resistance. The reason for this is not clear. We postulate that when healthcare is being delivered predominantly in the private sector, there are less controls and supervision of what is being done. This then may mean that there are fewer controls on broad-spectrum agents, the length of time of drug therapy and the volumes used.

We postulate that when Quality of Governance is poor, then there are likely to be less effective controls of antibiotic use (not only in people but in the animal sector). Thus, not only will more antibiotic resistant bacteria develop but the spread of these resistant bacteria will also be easier. This is because there will be less supervision and enforcement of laws that cover issues, not only of human medicine, but also of food and water safety. Reducing antimicrobial resistance requires a policy mix aimed at lowering antibiotic usage in people and, perhaps even more importantly, developing better controls on corruption.

Surprisingly, our results suggest that antibiotic resistance in the previous period does not affect present resistance. This implies that previously high levels of antimicrobial resistance in a country are not insurmountable barriers to future improvements.

The data we have shown only pertain to Europe. This is because this is only region where good data are available from multiple countries. Europe has data not only on the issues of government effectiveness and rule of law etc, but more importantly on the magnitude and extent of antimicrobial usage and resistance levels in serious bloodstream infections caused by common bacteria such as S. aureus and E. coli. We suspect that these same issues will pertain worldwide (e.g. corruption). In developing countries, they are likely to be much more of a factor, and quality of governance will likely explain why antibiotic resistance is so much higher in these countries than in most countries in Europe. The fact that these data are publicly available for Europe, suggest that issues of governance are also much better in Europe than in most of the rest of the world.

To our knowledge, this is the first cross-national examination of the determinants of antibiotic resistance. Our results, based on a systematic econometric analysis using a panel data set consisting of 29 European countries over the time period 1998–2010, suggest that governance is an even more important determinant of antibiotic resistance than antibiotic usage in people. The estimated impact of governance is large and highly statistically significant, using state-of-the-art econometric methodologies that pay particular attention to issues of endogeneity. The results are robust to the use of alternative estimation methods.

There are, however, limitations in our study. One of our main explanatory variables, “quality of governance” (*GOV*) is based on subjective assessment by experts. This variable comes from the International Country Risk Guide developed by the Political Risk Services Group [26], and measures the quality of governance in terms of the control of corruption. It is primarily meant to be a guide for foreign investors but has been commonly used in examining the impact of the quality of governance on various facets of general economic performance among countries [25, 27, 29]. Despite the potential bias involved in the construction of subjective indices, the fact that this indicator is widely purchased by investors for substantial sums of money shows a revealed preference for it [28].

We acknowledge that the issue of antibiotic resistance and how it develops and spreads is complex and our results may not be generalizable to other settings. Antibiotic resistance involves factors both in the human and agricultural sectors and the environment [33] and we have not been able to include all these factors as parameters in this study because adequate data are lacking. Antibiotic usage in food animals is one very important parameter that hopefully can in the future be examined when adequate data become available for all these countries. Another parameter for which there are no data available is the usage of the amounts of antibiotics in children. Travel is also a factor than can affect the level of resistant bacteria carried by people although in most it tends not to be persistent [34]; however, we have used an estimation methodology which takes into account the possible bias resulting from the omission of these variables. Despite limitations, we believe our data strongly suggest that the issue of antimicrobial resistance in human pathogens is not just related to the volume of antibiotic usage in people but other important social factors such as corruption and effective governance. Overall, the better any country was with respect to these factors, the lower were the resistance rates in bacteria causing serious and life-threatening infections there. These are important issues, which call for further research in other global jurisdictions.

Our findings have important policy implications. Just as a sizable literature has convincingly demonstrated that the quality of institutions (governance) is the fundamental determinant of economic growth—our results suggest that improving governance could be similarly fundamental in confronting the issue of antibiotic resistance. Improved governance at the national level is likely to imply better practices in the health sector, including controls and supervision of antibiotic use. It is also likely to lead to healthier outcomes in a whole array of other related areas that are intertwined with the issue of antibiotic resistance, such as the agriculture sector, food and water safety.

## Supporting Information

S1 Table Data used in the empirical analysis. (XLSX)
